# *In Vitro* Effect of Mitochondria-Targeted Triphenylphosphonium-Based Compounds (Honokiol, Lonidamine, and Atovaquone) on the Platelet Function and Cytotoxic Activity

**DOI:** 10.3389/fphar.2022.893873

**Published:** 2022-05-11

**Authors:** Héctor Montecino-Garrido, Diego Méndez, Ramiro Araya-Maturana, Juan Pablo Millas-Vargas, Sergio Wehinger, Eduardo Fuentes

**Affiliations:** ^1^ Department of Clinical Biochemistry and Immunohematology, Thrombosis Research Center, MIBI: Interdisciplinary Group on Mitochondrial Targeting and Bioenergetics (ACT210097), Medical Technology School, Faculty of Health Sciences, Universidad de Talca, Talca, Chile; ^2^ Instituto de Química de Recursos Naturales, MIBI: Interdisciplinary Group on Mitochondrial Targeting and Bioenergetics (ACT210097), Universidad de Talca, Talca, Chile

**Keywords:** platelet, cytotoxicity, antiplatelet, triphenylphosphonium, mitochondria

## Abstract

**Introduction:** Obtaining triphenylphosphonium salts derived from anticancer compounds to inhibit mitochondrial metabolism is of major interest due to their pivotal role in reactive oxygen species (ROS) production, calcium homeostasis, apoptosis, and cell proliferation. However, the use of this type of antitumor compound presents a risk of bleeding since the platelet activation is especially dependent on the mitochondrial function. In this study, we evaluated the *in vitro* effect of three triphenylphosphonium-based compounds, honokiol (HNK), lonidamine (LDN), and atovaquone (ATO), on the platelet function linked to the triphenylphosphonium cation by a lineal 10-carbon alkyl chain and also the decyltriphenylphosphonium salt (decylphos).

**Methods:** Platelets obtained by phlebotomy from healthy donors were exposed *in vitro* to different concentrations (0.1–10 μM) of the three compounds; cellular viability, exposure of phosphatidylserine, the mitochondrial membrane potential (∆Ψm), intracellular calcium release, and intracellular ROS generation were measured. Platelet activation and aggregation were induced by agonists (adenosine diphosphate, thrombin receptor-activating peptide-6, convulxin, or phorbol-12-myristate-13-acetate) and were evaluated by flow cytometry and light transmission, respectively.

**Results:** The three compounds showed a slight cytotoxic effect from 1 μM, and this was concomitant with a decrease in ∆Ψm and intracellular calcium increase. Only ATO produced a modest but significant increase in intra-platelet ROS. Also, the three compounds increased the exposure to phosphatidylserine in platelets expressed in platelets positive for annexin V. None of the compounds had an inhibitory effect on the aggregation or activation markers of platelets stimulated with three different agonists. Similar results were obtained with decylphos.

**Conclusion:** Triphenylphosphonium derivatives showed slight platelet toxicity below 1 μM, probably associated with their effect on ∆Ψm and exposure to phosphatidylserine, but no significant effect on platelet activation and aggregation, making them an antitumoral alternative with a low risk of bleeding. However, future assays on animal models and human trials are required to evaluate if their effects with a low risk for hemostasis are replicated *in vivo*.

## Introduction

Mitochondria are fundamental organelles for the functioning of any type of cell since the vast majority of cellular processes and pathways are related in one way or another to the capacity of generating energy, regulating redox systems, generating metabolites, and controlling cell death (Kang and Pervaiz, 2012; Medini et al., 2020).

Within each mitochondrion, there are five complexes responsible for the passage of electrons and for oxidative phosphorylation, and the failure of functioning of these complexes can cause a lack of ATP, an excess of cytoplasmatic calcium, and/or an increase in reactive oxygen species, which can affect muscles, pancreas, brain, heart, and many other organs, leading to various heart, neurological, and endocrine problems (Dudkina et al., 2010).

Therefore, the drugs aimed at improving mitochondrial function are widely used in the treatment of many pathologies, as is the case of coenzyme 10, vitamin complexes B1 and B2, alpha-lipoic acid, l-carnitine, creatine, and L-arginine (Zielonka et al., 2017).

On the other hand, some diseases cause an abnormal increase in the mitochondrial function, which leads to high energy expenditure, change in the mitochondrial pathways, and production of excess reactive oxygen species (ROS), as seen in the development of diabetes and cancer (Williams and Caino, 2018; Dai and Jiang, 2019).

In direct relation to this, there are some compounds that target mitochondria and are capable of inhibiting the functioning of one or more of the mitochondrial complexes. The lipophilic triphenylphosphonium cation is capable of accumulating in the mitochondrial matrix, proportionally to the difference in the potential present within the mitochondria, which makes it especially interesting to use in overactive mitochondria with increased potential, such as tumor cells (Reily et al., 2013).

**FIGURE 1:**
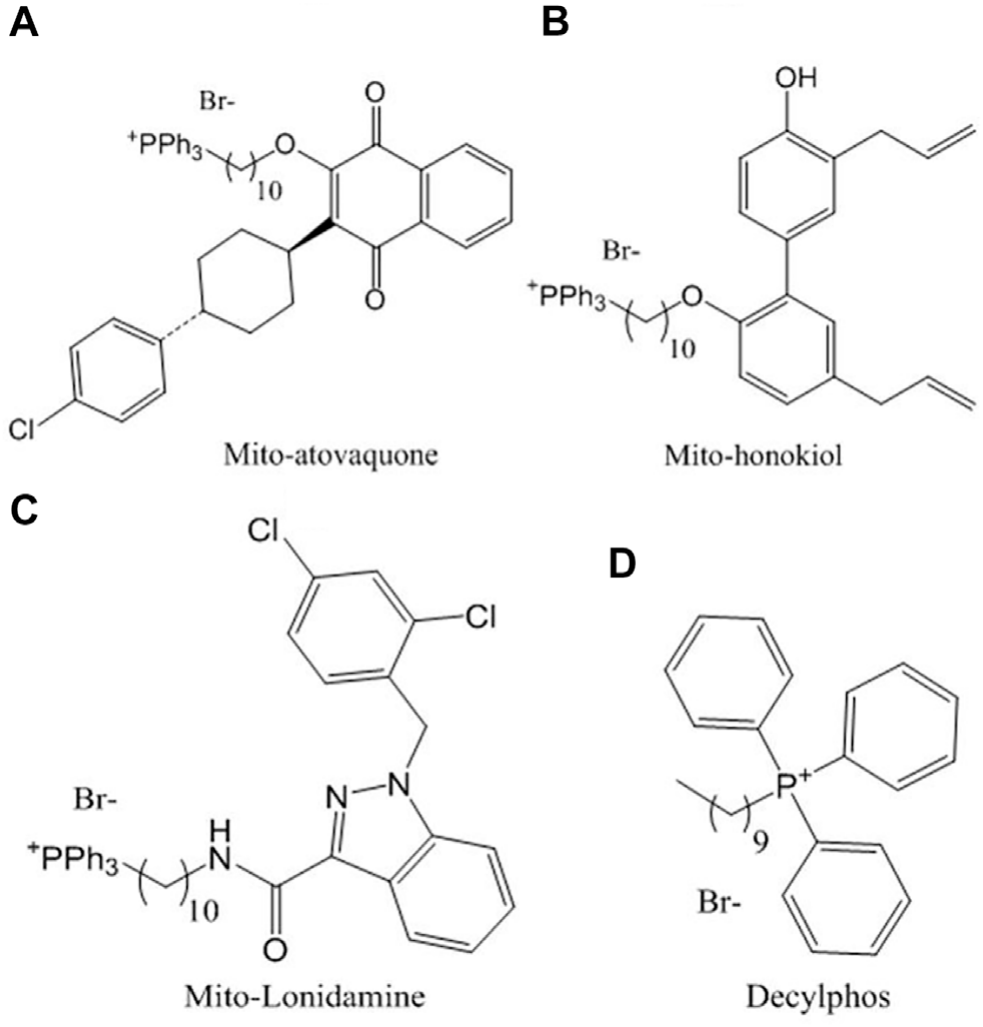
Chemical structures of the compounds conjugated with triphenylphosphonium studied in this work: **(A)** ATO, **(B)** HNK, **(C)** LND and **(D)** decylphos.

Among the compounds with the best antitumor activity is mito-atovaquone (ATO) (Figure 1A), a derivative of the compound atovaquone, a drug used for the treatment of malaria and has been reported to act as a competitive inhibitor on cytochrome bc1 complex or complex III. Its potency as an antitumor drug is increased when it is linked with the lipophilic triphenylphosphonium cation by acyl chains of different lengths. Those with 4 and 10 carbons inhibit both complexes I and III, while those with 12 and 16 carbons inhibit only complex I (Cheng et al., 2020).

Mito-honokiol (HNK) (Figure 1B) is a compound derived from honokiol lignan isolated from magnolia leaves, with a potency 100 times greater than its parent honokiol, with the ability to inhibit cell proliferation and mitochondrial complex I in metastatic mouse tumors favoring apoptosis and decreasing cell proliferation (Pan et al., 2018).

Finally, mito-lonidamine (LND) (Figure 1C) is another mitochondrial-targeted compound with a potency 100 times greater than the antiglycolytic drug from which it was synthesized (lonidamine); its ability to reduce cellular viability, growth, and progression is contrasted with the lack of toxicity at 50 times the effective dose for 8 weeks in murine models (Cheng et al., 2019).

When considering the use of these compounds in humans, one of the major concerns is the risk of bleeding, mainly due to the interaction of blood cells responsible for hemostasis: the platelets. It is known that these cells are especially dependent on their mitochondrial function, and an alteration in their metabolism could inhibit metabolic pathways and, at the same time, produce a significantly increased risk of bleeding (Baaten et al., 2018). For this reason, it is of particular importance to study the effects of these antitumor compounds on the functioning of platelets, to better consider the risks, and favor the development of therapies that reduce the possibility of bleeding.

## Materials and Methods

### Mitochondria-Targeted Compounds

The compounds conjugated with triphenylphosphonium ATO (Figure 1A), HNK (Figure 1B), and LND (Figure 1C) were provided by Dr. Balaraman Kalyanaraman’s lab, Medical College of Wisconsin, United States. Decylphos (Figure 1D), as already described (Eyles and Trippett, 1966), was obtained by nucleophilic substitution of decyl bromide as follows: To a 10 ml Monowave 50 (Anton Paar chemical reactor CEM) process vial equipped with a magnetic bar, a mixture of 1 equivalent (500 mg) of decyl bromide, 1.25 equivalent triphenylphosphine (1.45 g), and 5 ml of toluene was heated at 150°C for 30 min. After that, the phosphonium salt deposited at the bottom of the tube was thoroughly washed with toluene to obtain 308 mg (29%) of the phosphonium salt decylphos. Its purity was assessed by thin-layer chromatography using CH_2_Cl_2_/MeOH (20:1) as an eluent. Then, it was dried under a high vacuum to yield a viscous oil. All compounds were resuspended in dimethyl sulfoxide (DMSO) (Méndez et al., 2020a).

### Human Platelets

The platelets were obtained by phlebotomy from healthy donors (2 weeks without drugs) who had previously accepted informed consent, as previously reported (Méndez et al., 2020a). Briefly, whole blood was obtained with an acid-citrate-dextrose (ACD) solution (proportion 4:1 v/v) and centrifuged at room temperature for 12 min × 200 g to collect platelet-rich plasma (PRP). Then, the PRP was centrifuged for 8 min × 900 g. The platelet pellet was suspended in calcium-free Tyrode’s buffer/ACD (proportion: 5:1 v/v) and this was centrifuged again for 8 min × 900 g; then, the platelets were resuspended t in calcium-free Tyrode’s buffer and adjusted to the assays with a hematological counter (Mindray BC-3000 Plus, Japan) and used within 3 h (Monroy-Cárdenas et al., 2020). The discard of pre-activation of platelets during preparation is shown in Supplementary Figure S1. Thus, the expression of P-selectin is similar between PRP and washed platelets, while the addition of ADP and TRAP-6 increases similarly its expression on platelets. Also, Supplementary Figure S1 shows that the mouse IgG1-PE isotype control did not induce a non-specific binding.

### Cytotoxic Activity by LDH

The washed platelets (200 × 10^6^ platelets/ml) were incubated with each compound (0.1, 0.5, 1, 2, and 10 μM) for 10 min at 37°C. DMSO was used as a vehicle. The platelets were then centrifuged at 900 g for 8 min to obtain the supernatant, which was evaluated with a lactate dehydrogenase (LDH) cytotoxicity kit (Cayman Chemical, Ann Arbor, MI, United States) (Rywaniak et al., 2015). The platelets incubated with a 10%Triton X-100 solution were used as a positive control of cytotoxicity.

### Procoagulant Activity (Externalization of Phosphatidylserine)

The washed platelets (200 × 10^6^ platelets/ml) were incubated with each compound (1 and 2 μM) for 10 min at 37°C. DMSO was used as a vehicle. After that, the externalization of phosphatidylserine (procoagulant activity) was determined by Annexin V FITC (Annexin V-FITC Apoptosis Staining/Detection Kit, ABCAM) binding to phosphatidylserine and measured with a BD FACS Lyric flow cytometer (BD Biosciences, United States). The fraction (%) of positive platelets for annexin V FITC was evaluated (Méndez et al., 2021). As a positive control of procoagulant activity, platelets were stimulated with convulxin 10 ng/ml and TRAP-6 5 μM.

### Cell Viability by Calcein-AM

The washed platelets (200 × 10^6^ platelets/ml) were incubated with each compound (0.1, 0.5, 1, 2, and 10 μM) for 10 min at 37°C. DMSO was used as a vehicle. After that, viability was determined using a BD FACS Lyric flow cytometer (BD Biosciences, United States) with Calcein-AM (Rywaniak et al., 2015). The fraction (%) of platelets Calcein-negative in the subpopulation of CD61^+^ were recognized as non-viable cells. Triton X-100 (0.5%) was used as an inducing control of cell damage (Méndez et al., 2020a).

### Mitochondrial Membrane Potential (∆Ψm)

The mitochondrial membrane potential was determined using the potentiometric probe tetramethylrhodamine methyl ester perchlorate (TMRM) 100 nM and analyzed with a BD FACS Lyric flow cytometer (BD Biosciences, United States). Each compound was evaluated at 1 and 2 μM on washed platelets (50 × 10^6^ platelets/ml), and, 1 μM carbonyl cyanide-p-trifluoromethoxyphenylhydrazone (FCCP) was used as a control of mitochondrial depolarization (Méndez et al., 2021).

### Intraplatelet ROS Levels

ROS production was determined in washed platelets (50 × 10^6^ platelets/ml) using dihydroethidium (DHE) 10 μM and analyzed with BD FACS Lyric flow cytometer (BD Biosciences, United States). The labeled platelets were pre-incubated with each compound (1 and 2 μM) and then incubated for 30 min at 37°C. Antimycin A 20 μM was used as a positive control for increased ROS (Méndez et al., 2020a).

### Intraplatelet Calcium Levels by Flow Cytometry

The washed platelets (200 × 10^6^ platelets/ml) were mixed with Fluo-3-AM (0.44 μM). Labeled platelets were incubated for 30 min at room temperature in the dark. Subsequently, they were diluted to a count of 50 × 10^6^ platelets/ml and incubated for 10 min at 37°C with vehicle (0.4% DMSO), positive control, or compounds (1 and 2 μM), and then the reading was performed in a BD FACS Lyric flow cytometer (BD Biosciences, United States). FCCP 1 μM was used as a positive control for the increase in intracellular calcium. The effect of the compounds on cytosolic calcium mobilization was normalized relative to the control (Méndez et al., 2020b). To evaluate the calcium curve, measurements were made at 0.5, 5, and 10 min (Supplementary Figure S2). Thus, Supplementary Figure S2 shows that at 30 s after the addition of the compounds (ATO, HNK, and LND) or FCCP, the intracellular calcium begins to increase. This increase is progressive between 5 and 10 min. Meanwhile, the intraplatelet calcium level for the vehicle was stable.

### Intraplatelet Calcium Levels by Fluorescence Microscopy

The washed platelets (200 × 10^6^ platelets/ml) were mixed with Fluo-3-AM (0.8 μM). The labeled platelets were incubated for 30 min at room temperature in the dark. Subsequently, platelets were diluted to a count of 50 × 10^6^ platelets/ml and incubated for 10 min at 37°C with vehicle (DMSO 0.4%) or compounds (1 and 2 μM). FCCP (1 μM) was used as a positive control for the increase in intracellular calcium. Intraplatelet calcium was measured by fluorescence microscopy (AXIO Examiner Z. 1, Zeiss, Oberkochen, Germany). Briefly, 10 μl of FLUO-3 AM-labeled platelets were spotted onto a slide with the collagen film and heat-fixed as previously described (Méndez et al., 2020a). The 63x objective of the COLIBRI system was used to 470 nm (blue light) at 80% intensity. Subsequently, the mean fluorescence intensity (MFI) of the photographs obtained was quantified with ImageJ software (Schneider et al., 2012), where it was segmented with the “threshold” tool and the MaxEnthropy method. Then, a selection based on the thresholding was created, and the “mean gray value” was measured from the selected cells. Representative images are shown in Supplementary Figure S3.

### Platelet Activation (P-Selectin and CD63)

The effect of each compound (1 and 2 μM) on P-selectin (CD62P) and CD63 platelet expression (%) were measured with a BD FacsLyric flow cytometer (Fuentes et al., 2014) in washed platelets or PRP incubated with anti-CD62P-PE or anti-CD63-PE for 30 min at room temperature. As a general platelet marker, anti-CD61-FITC (platelet glycoproteins IIIa) antibody was used (Méndez et al., 2020b).

### Platelet Aggregation

Platelet aggregation was evaluated by light transmission (AggRAM—Helena Biosciences) (Méndez et al., 2020b). The washed platelets or PRP (200 × 10^6^ platelets/ml) were used. The washed platelets (with CaCl_2_ 2 mm) or PRP were incubated with the compound (1 and 2 μM) for 5 min at 37°C. Following this, platelet aggregation was stimulated by adenosine diphosphate (ADP) in PRP, and convulxin 5 ng/ml, TRAP-6 5 μM, or phorbol 12-myristate 13-acetate (PMA) 100 nm in washed platelets. Platelet aggregation was measured for 5 min at 37°C.

### Statistical Analysis

Data were obtained from experiments from at least three healthy volunteers, analyzed using Prism 9.0 software (GraphPad Inc., San Diego CA, United States), and expressed as mean ± standard error of the mean (SEM). The differences between groups were analyzed using a one-way analysis of variance (ANOVA) and the Bonferroni post hoc test. *p* values <0.05 were considered statistically significant.

## Results

### Cytotoxic Effect and Procoagulant Activity (Exposure of Phosphatidylserine)

**FIGURE 2:**
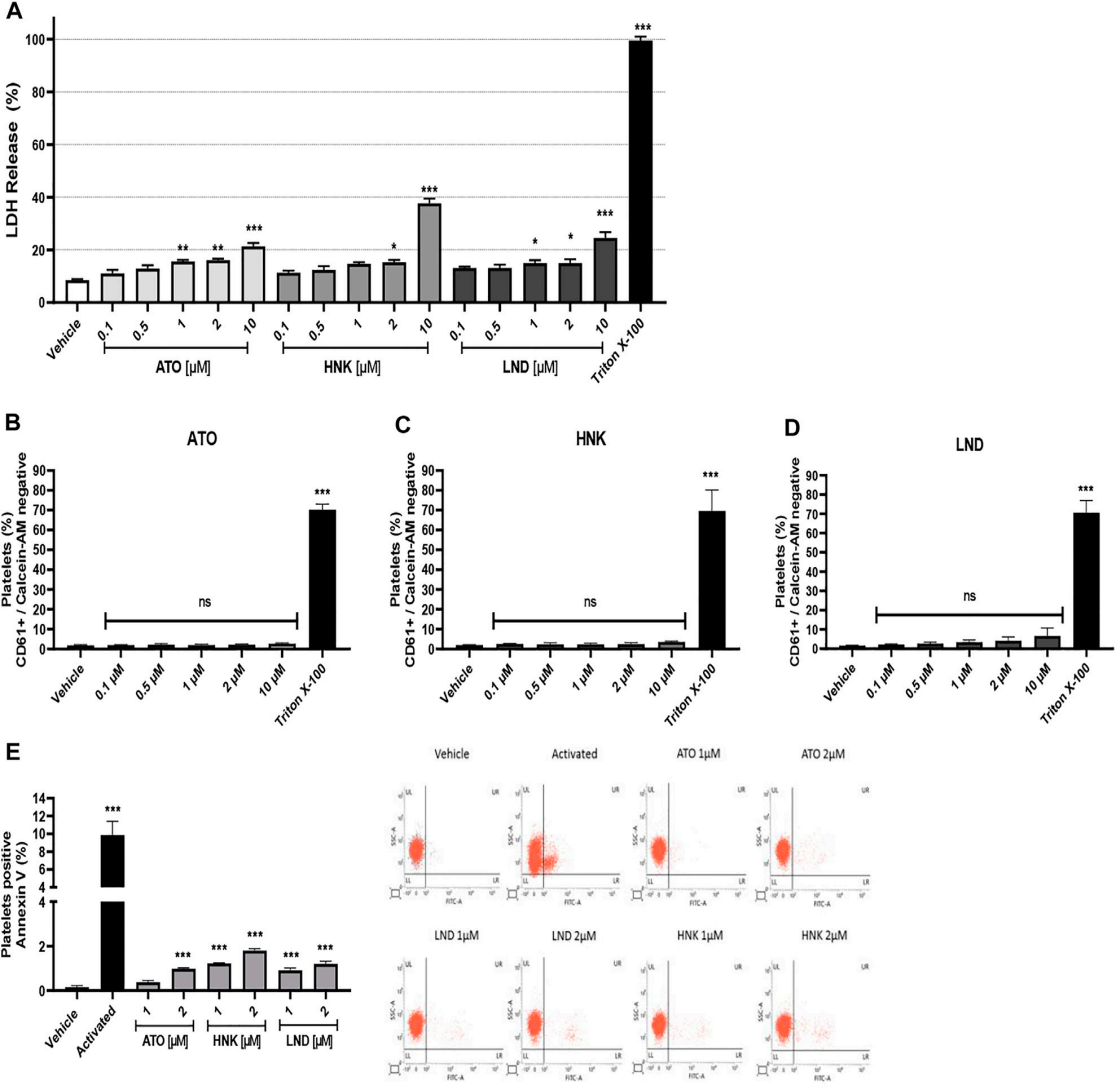
Cytotoxic effect and procoagulant activity (exposure of phosphatidylserine). **(A)** LDH release from platelets, **(C–E)** platelet viability studied with Calcein-AM, and **(E)** procoagulant activity by annexin V binding and representative dot plots from flow cytometry. The results were obtained by LDH release from the supernatant on washed platelets and evaluated by flow cytometry. For viability, platelets were stained with CD61 antibody and Calcein-AM, and the subpopulation of CD61+/Calcein-AM negative was considered non-viable as it was analyzed in terms of change in the mean fluorescence intensity from the vehicle. For procoagulant activity, the washed platelets were incubated with Annexin V FITC; the positive control corresponds to platelets activated with convulxin and TRAP-6. The results were expressed as mean ± SEM. Vehicle: DMSO 0.4%. The statistical analysis was performed using a one-way analysis of variance (ANOVA) and the Bonferroni post hoc test. ∗*p* < 0.05, ∗∗*p* < 0.01, and ∗∗∗*p* < 0.001 vs. vehicle. ns: not significant.

The compounds were tested for the release of LDH and calcein-AM at concentrations of 0.1, 0.5, 1, 2, and 10 μM. It is observed that the compounds ATO and LND significantly increase the release of LDH at concentrations 1, 2, and 10 μM; in the case of the compound HNK, the release of LDH significantly increases only at concentrations 2 and 10 μM (Figure 2A). These results denote a slight cytotoxic effect, probably associated with an increase in the permeability of the plasmatic platelet membrane. The 10% Triton X-100 control was used as the maximum percentage of LDH release due to its detergent effect on washed platelets. To evaluate platelet viability, Calcein-AM was measured by flow cytometry. The results were expressed as the percentage of CD61+/Calcein-AM negative platelets (not viable). Statistical analysis shows a significant increase in non-viable platelets only for the 0.5% Triton X-100 control when compared with the vehicle (Figures 2B–D). These results together suggest that the compounds do not affect platelet viability *in vitro* but alter platelet membrane permeability at concentrations of 1 μM for ATO and LND and of 2 μM for HNK.

The effect of the compounds (ATO, HNK, and LND) on the procoagulant activity (exposure of phosphatidylserine) in platelets was evaluated by flow cytometry. It was observed that the compound ATO 2 μM significantly increases the percentage of platelets positive for annexin V (Figure 2E). In the same way, the HNK and LND compounds at both concentrations tested (1 and 2 μM) significantly increase the percentage of the annexin V positive population when compared to the vehicle. As a positive control of procoagulant activity, the combination of convulxin and TRAP-6 was used to stimulate platelets, which showed a significant increase in exposure to phosphatidylserine when compared to the vehicle. Also, Supplementary Figure S4 shows that the effect of compounds (HNK and LND) on procoagulant activity is maintained in presence of convulxin. Meanwhile, in response to TRAP-6 alone or plus convulxin, no significant differences were observed by the compounds (ATO, HNK, and LND) on procoagulant activity.

### Effect on Platelet Mitochondria

**FIGURE 3:**
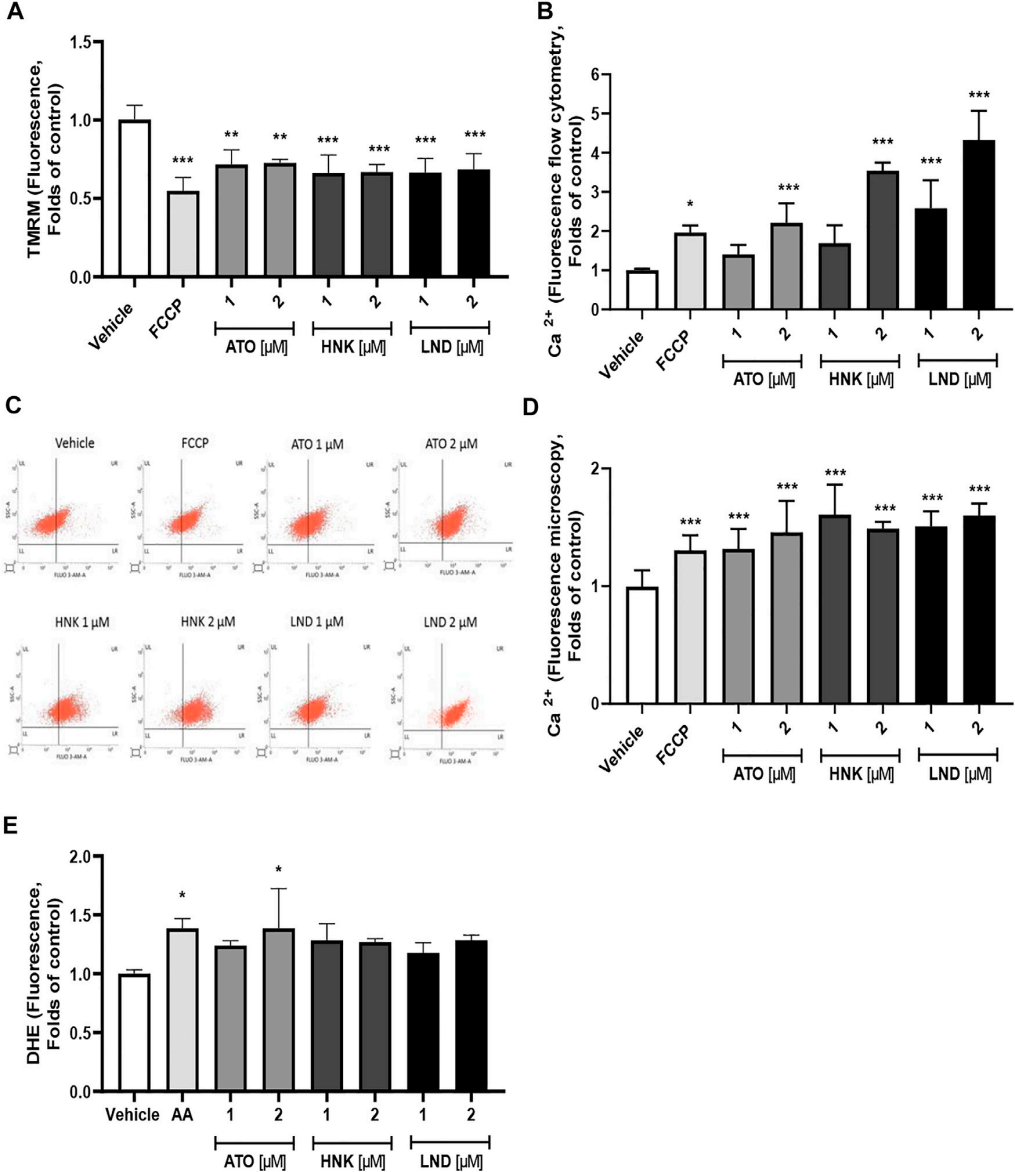
Effect of the compounds on the **(A)** mitochondrial membrane potential (∆Ψm), **(B–D)** intraplatelet calcium levels, and **(E)** intraplatelet ROS levels. The results were obtained by **(B,C)** flow cytometry and **(D)** fluorescence microscopy. **(B)** Representative dot plots from flow cytometry of intraplatelet calcium levels. Platelets were identified as CD61^+^ population and these CD61 expressions were analyzed in terms of change in the mean fluorescence intensity from the vehicle. FCCP was used as a positive control of mitochondrial depolarization; AA was used as a positive control of ROS induction. The results were expressed as mean ± SEM. Vehicle: DMSO 0.4%. The statistical analysis was performed using a one-way analysis of variance (ANOVA) and the Bonferroni post hoc test. ∗*p* < 0.05; ∗∗*p* < 0.01, and ∗∗∗*p* < 0.001 vs. vehicle.

The three compounds (ATO, HNK, and LND) at concentrations of 1 and 2 μM significantly decreased the mitochondrial membrane potential in washed platelets, as observed in Figure 3A. Regarding the effect of the compounds on intracellular calcium levels, a significant increase was observed with ATO, HNK, and LND (Figures 3B–D). This effect on calcium levels is associated with a decrease in the mitochondrial membrane potential because when depolarization occurs, calcium is released in response to mitochondrial stimulation. This phenomenon in the mitochondrial function is evident when using the FCCP control. As shown in Figures 3A,B, the membrane potential decreased while the levels of intra-platelet calcium were increased in a statistically significant way. Finally, as shown in Figure 3E, regarding intracellular ROS levels, only 2 μM ATO produced a significant amount of intra-platelet ROS to the control similar to 10 μM Antimycin A (ROS inducing control).

### Effect on Platelet Activation

**FIGURE 4:**
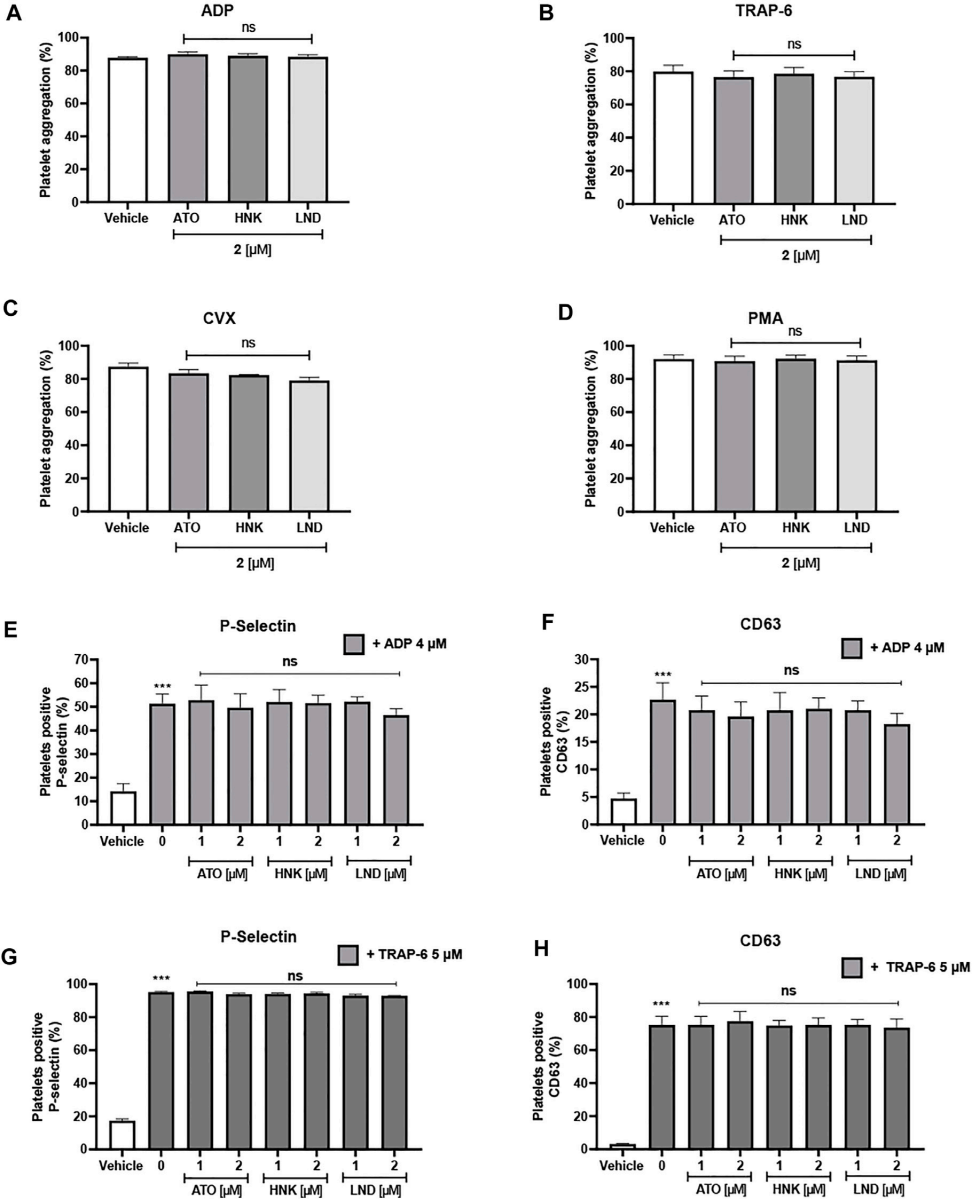
Lack of effect of the compounds upon platelet aggregation and platelet activation markers. Platelet aggregation is induced by **(A)** ADP, **(B)** TRAP-6, **(C)** convulxin, and **(D)** PMA. P-selectin expression **(E)** and CD63 expression **(F)** on platelet-rich plasma stimulated with ADP. P-selectin expression **(G)** and CD63 expression **(H)** on washed platelets stimulated with TRAP-6. Platelets were identified as CD61^+^ population and these CD61-expressions were analyzed in terms of the percentage of positive platelets to the activation marker. The results were expressed as mean ± SEM. Vehicle: DMSO 0.4%. The statistical analysis was performed using a one-way analysis of variance (ANOVA) and the Bonferroni post hoc test. ∗*p* < 0.05; ∗∗*p* < 0.01 and ∗∗∗*p* < 0.001 vs. vehicle, ns: not significant vs. 0 **(E–H)**.

Figure 4 describes the effect of these three compounds on platelet aggregation and activation As observed in Figures 4A–D, up to 2 μM concentration, none of the three compounds had any inhibitory effect on the aggregation of washed platelets stimulated with four different agonists (ADP, TRAP-6, convulxin, and PMA). When analyzing the effect on activation markers in platelet-rich plasma, it is observed that platelets stimulated with ADP significantly increase the expression of P-selectin and CD63 in the membrane, when compared to the vehicle (without agonist). When evaluating the effect of the compounds on ADP-induced activation, a significant decrease in the expression of the activation markers evaluated was not observed, which correlates with also the null effect on platelet aggregation in PRP (Figures 4E,F) or the washed platelets (Figures 4G,H).

**FIGURE 5:**
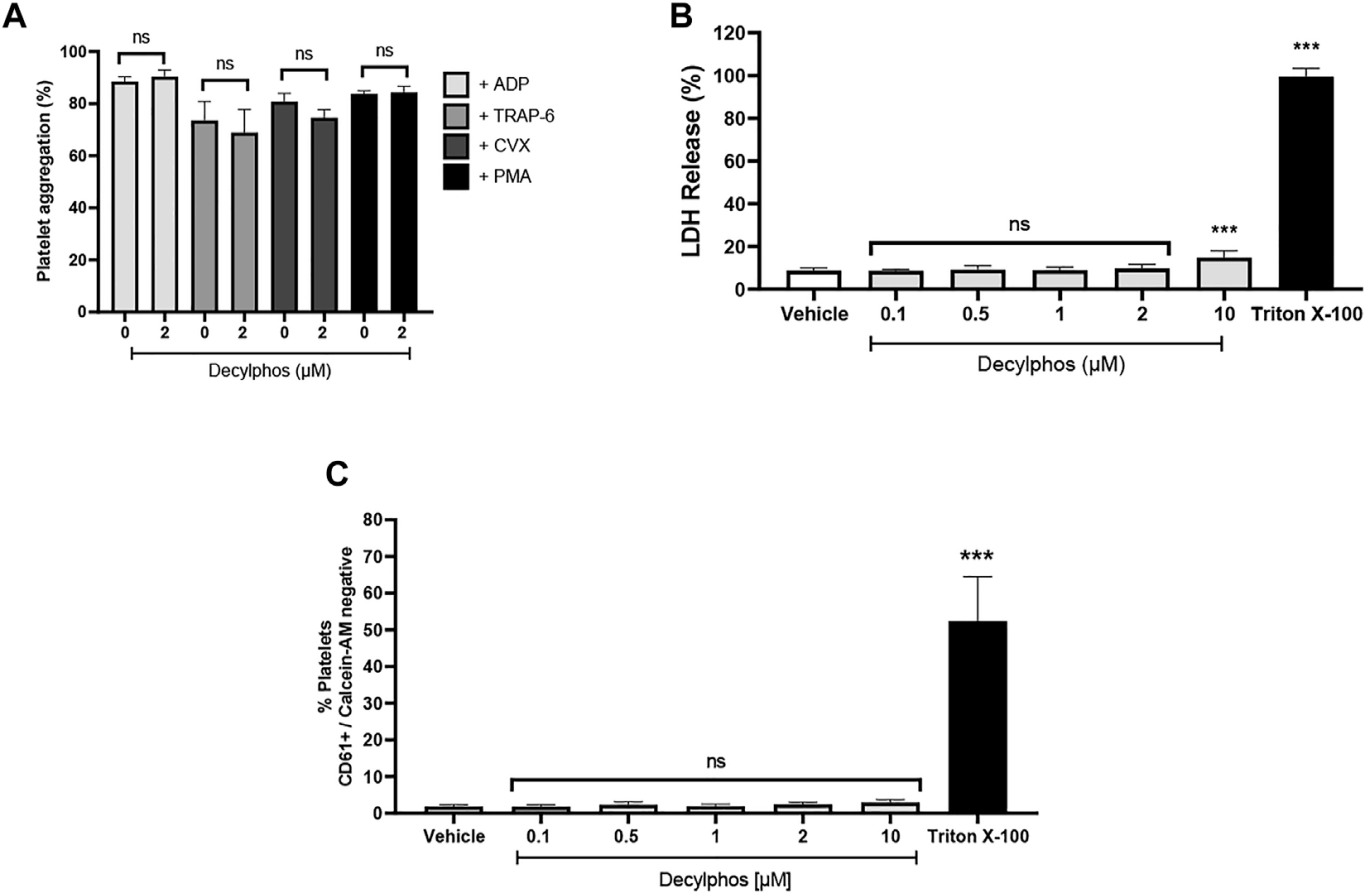
Effect of decylphos on platelet aggregation, cytotoxicity, and cell viability. **(A)** Platelet aggregation **(B)**, LDH release from platelets **(C)**, and platelet viability by Calcein-AM. The results were expressed as mean ± SEM. Vehicle: DMSO 0.4%. The statistical analysis was performed using a one-way analysis of variance (ANOVA) and the Bonferroni post hoc test. ∗∗∗*p* < 0.001 vs. vehicle.

Figure 5 shows the effect of the 10-carbon chain triphenylphosphonium cation (decylphos) on platelet aggregation, cytotoxicity, and viability. At 2 µM, decylphos shows no inhibitory effect on the platelet aggregation in PRP stimulated with ADP and washed platelets stimulated with TRAP-6, convulxin, and PMA (Figure 5A). In addition, it shows a significant increase in platelet cytotoxicity due to the release of LDH at a10 μM concentration (Figure 5B), when compared to the vehicle. Finally, it shows no effect on the viability of the washed platelets (Figure 5C). These results are similar to those obtained for the three compounds under study (ATO, HNK, and LND).

## Discussion

The use of compounds targeting the mitochondria is of major interest due to the accumulation of bioactive molecules in proximity to the electron transporter complex, affecting the mitochondrial bioenergetics and, in turn, the rest of cellular activities. In the case of tumor cells, the aim is to decrease the mitochondrial membrane potential, accompanied by tumor suppression (Zielonka et al., 2017). For this, the most efficient structure comes with a chain length of 10 carbons as was demonstrated in 2015 when Trnka et al. (2015) showed an increase in oxygen consumption and a decrease in the metabolic rate, proportional to the length of the triphenylphosphonium derivatives, emphasizing the effect of the compound decylphos, a molecule used in this research as a control to compare the effect of the conjugated compounds ATO, HNK, and LND.

Atovaquone was originally used in the treatment of malaria in its pure form as a complex III inhibitor. Because it removes ubiquinol from its active site (Birth et al., 2014), it can change the mitochondrial membrane potential; this property along with the accumulation granted by triphenylphosphonium was considered an excellent proposal to eradicate tumor cells (Fiorillo et al., 2016). This is arguably correct, looking at the increased efficiency as an inhibitor; it lowered the IC_50_ from 18.8 to 0.22 μM (Cheng et al., 2020).

Honokiol, as a complex I inhibitor, decreases ATP production and stimulates ROS production, reducing metastasis in tumor cells (Pan et al., 2016), which makes it an excellent candidate to conjugate with triphenylphosphonium; this is evident in the confluence tests, where the pure honokiol has an IC_50_ of 27 µM, while mito-honokiol (HNK) has an IC_50_ of 0.26 µM (Pan et al., 2018).

Finally, lonidamine, a long-known anticancer drug, inhibits the fumarate and malate synthesis and the succinate-dependent respiration produced by complex II (Guo et al., 2016). The effect of this compound, when linked with a triphenylphosphonium cation, reduces the IC_50_ from 208 to 0.69 μM in A549 human adenocarcinoma cells, a considerable leap in the antitumoral capacity of the drug.

This difference in tumor cell activity is explained as described by Cheng et al. (2020) since a 100-fold lower concentration of the conjugate compound is required to obtain the same inhibitory effect. This ease of killing tumor cells means a double-edged sword because it could affect healthy cells with a high dependence on their mitochondrial capacity, making them highly reactive or inhibiting their activity (Fuentes et al., 2019).

In agreement with the studies published by other authors, platelets showed a significant decrease in the mitochondrial potential at 1 and 2 μM (Figure 3A), without an increase in ROS production except for 2 μM ATO (Figure 3E). On the other hand, it only produced a significant increase in calcium over 2 μM in ATO and HNK, while LND produced a significant increase in both, 1 and 2 μM (Figure 3B), that increased with time (Supplementary Figure S2). The results of the increase in the intraplatelet calcium induced by FCCP as measured by Fluo-3 (Figure 3B) suggest that a decrease in the mitochondrial membrane potential (Figure 3A) is caused by a proton leak, and an increased membrane permeability promotes the release of calcium directly from mitochondria. However, we cannot discard that the calcium release induced by the compounds could also participate in other intracellular sources, such as the dense tubular system (DTS) or lysosome-related organelles (LROs).

By observing the effect of these compounds on platelet markers, it was verified that despite the fact that the inhibition of mitochondrial bioenergetics can lead to a decrease in activation and aggregation as was postulated by Barile et al. (2012), platelet aggregation was not affected by any compound either with the agonists ADP in PRP, or TRAP-6, convulxin, and PMA in washed platelets. The expression of P-selectin or CD63 was not affected at these concentrations when stimulated with 4 μM ADP. Although there was a small decrease in platelet activation depending on the dose, this was not significant, which shows resilience due to the metabolic plasticity in the platelet (Ravi et al., 2015).

The mitochondrial membrane potential drives the transport of inward cations and outward anions, and it has been demonstrated to exert important effects on cell viability. In this context, we showed that compounds (ATO, HNK, and LND) generate mitochondrial disruption, which is closely associated with platelet phosphatidylserine exposure and regulation of life span (Choo et al., 2017). Thus, when evaluating the safety range in which ATO can be used, the concentration at which cytotoxicity (verified by LDH release) significantly increased was five times higher than the IC_50_ reported for tumor cells (Figure 2A), while with the Calcein-AM-based assay, it did not show a significant decrease in viability even up to 10 μM (Figures 2B–D). A significant increase in cytotoxicity was observed by LDH release, at concentrations closer to 2 μM, showing a difference of 7%–8% normalized to the control with Triton X-100 (Figure 2A).

The disparity in the sensibility of the techniques could be due to the nature of the measuring device since the LDH assay uses the leaked LDH enzyme as an indirect measure of the global membrane permeability and it can detect smaller damage in a large group of cells; instead, the lack of calcein-AM inside a cell as a measure of dead cells in a flow cytometer requires the total depletion of the dye in single cells to be considered as truly dead.

As described in the literature, the accumulation of triphenylphosphonium-conjugated compounds follows a 10-fold increment for every 61.5 mV of the membrane potential with normal mitochondria, concentrating up to 15 times more than the cytosolic fraction (Murphy, 2008). The ability of healthy mitochondria to concentrate lipophilic cations according to their potential made it possible to measure reliably the differences in the voltage using other triphenylphosphonium cations such as 18F-fluorophenyl (Kim et al., 2012). This effect can explain the disparity between toxicity to tumor cells and toxicity to platelets, as the high mitochondrial potential that tumor cells possess, compared to a normal cell, does increase the accumulation of the compound inside the matrix (Creed and McKenzie, 2019) and, especially, those with a higher degree of malignancy, causing higher mitochondrial potentials in the tumor cells studied (Ye et al., 2011; Zhang et al., 2015).

It has been postulated in previous studies that the carbon chain length can favor the entry of lipophilic cations and increase both the rate of entry and its toxicity since the increase in carbons in an aliphatic chain produces an increase in hydrophobicity, controlling the membrane binding and lipid bilayer infiltration (Asin-Cayuela et al., 2004). This can make it easier for the conjugated drug to access the active sites in the respiratory complexes (James et al., 2007), but it comes with its disadvantages since it was observed that triphenylphosphonium derivatives with a chain length of 10 have increased toxicity close to 10 μM, as shown by the tests with MitoQ based on frog epithelial cells (Fock et al., 2018) or even platelets (Méndez et al., 2020a).

In this sense, we use the compound decylphos, which is composed of an aliphatic chain of 10 carbons linked to the triphenylphosphonium cation, as a control for the effect of the linker on platelets and showed slight toxicity at 10 µM in the LDH release assays as it had on the other compounds tested, but on a smaller scale than that shown by the mitochondrial-targeted compounds. This added to the fact that neither platelet aggregation nor the expression of activation markers, such as P-selectin or CD63, was affected using decylphos, showing that part of the cytotoxicity can be modulated by controlling the size and hydrophobicity of the linker.

## Conclusion

The three triphenylphosphonium-based compounds with an alkyl linker of 10 carbons showed a slight increase in platelet toxicity by LDH release assay, without a significant decrease in cell viability as seen by the number of platelet CD61(+)/Calcein-AM(−). This effect could be related to an increase in membrane permeability and proton leak caused by the length of the linker and hydrophobicity upon the mitochondrial potential and procoagulant activity. The decrease in the mitochondrial potential and the dose-dependent increase in intracellular calcium did not relate to ROS production, suggesting a detergent-like effect upon the mitochondria and a platelet’s metabolic plasticity resilient enough to buffer the effect upon the platelet function, judging by the expression of P-selectin and CD63, both markers of granule secretion, as well as the aggregation with ADP, TRAP-6, convulxin, and PMA. The low toxicity and lack of reactivity *in vitro* make them an antitumoral alternative with a low risk of bleeding. However, future assays on animal models and human trials are required to evaluate if their effects with a low risk for hemostasis are replicated *in vivo*.

## Data Availability

The original contributions presented in the study are included in the article/Supplementary Material, further inquiries can be directed to the corresponding author.
