# Supplementary material for: In Vitro Effect of Mitochondria-Targeted Triphenylphosphonium-Based Compounds (Honokiol, Lonidamine, and Atovaquone) on the Platelet Function and Cytotoxic Activity
Source: Front Pharmacol. 2022 May 11;13:893873. doi: 10.3389/fphar.2022.893873 (PMC9130573; doi:10.3389/fphar.2022.893873)
Supplement: Supplementary file 1 [file DataSheet1.docx]

**SUPPLEMENTARY INFORMATION**

***The in vitro effect of mitochondria-targeted triphenylphosphonium-based compounds (honokiol, lonidamine, and atovaquone) on platelet function and cytotoxic activity.***

**Figure S1**

**Figure S1.** Discard of pre-activation of platelets during preparation. (A) P-selectin expression in platelet-rich plasma (PRP) and washed platelets; (B) P-selectin expression in activated platelets and (C) Mouse IgG1 Isotype PE Control. The results were measured by flow cytometry. Platelets were identified as CD61+ population and values are expressed as the percentage of positive platelets. The results were obtained from three independent assays (mean+SEM). Vehicle: DMSO 0.4%. The statistical analysis was performed using a one-way analysis of variance (ANOVA) and the Bonferroni post hoc test. ***p<0.001 vs vehicle; ###p<0.001 vs WP. ns: not significant; PRP: platelet-rich plasma; WP: washed platelets.

**Figure S2**

**Figure S2.** The curve of intraplatelet calcium levels. The results were obtained by flow cytometry. Platelets positive FLUO-3 AM were analyzed in terms of change in mean fluorescence intensity from the vehicle. FCCP was used as a positive control of mitochondrial depolarization. The results were obtained from three independent assays (mean+SEM). Vehicle: DMSO 0.4%.

**Figure S3**


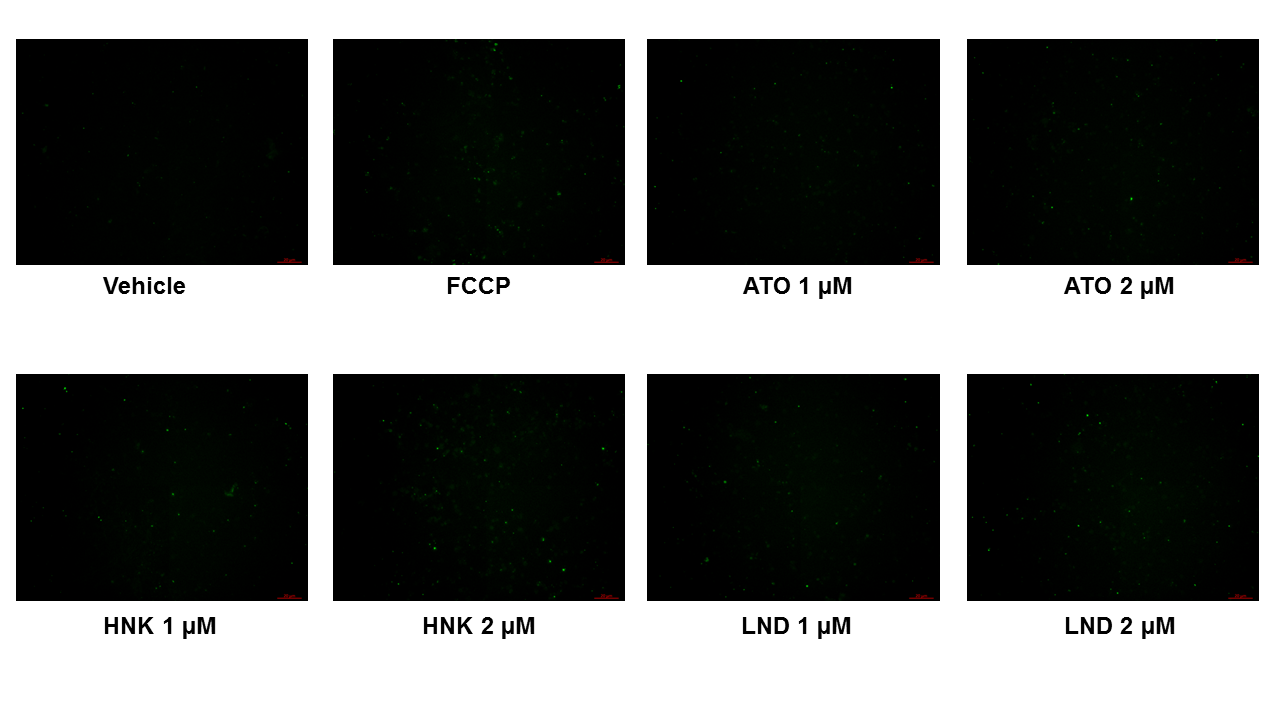


**Figure S3.** Representative images of intraplatelet calcium levels by fluorescence microscopy.

**Figure S4**

**Figure S4.** Procoagulant activity. (A) Stimulated with convulxin (CVX); (B) stimulated with TRAP-6 and (C) stimulated with CVX plus TRAP-6. Washed platelets were incubated with Annexin V-FITC. The results are reported as the percentage of positive platelets for Annexin V FITC. The results were obtained from three independent assays (mean±SEM). Vehicle: DMSO 0.4%. The statistical analysis was performed using a one-way analysis of variance (ANOVA) and the Bonferroni post hoc test. #p<0.05, ##p<0.01 and ###p<0.001 vs vehicle. *p<0.05 and ***p<0.001 vs 0 (activated control). CVX: Convulxin.
